# Crystal structure of 5-chloro-3-(4-fluoro­phenyl­sulfin­yl)-2,4,6-trimethyl-1-benzo­furan

**DOI:** 10.1107/S1600536814019229

**Published:** 2014-09-03

**Authors:** Hong Dae Choi, Uk Lee

**Affiliations:** a Department of Chemistry, Dongeui University, San 24 Kaya-dong, Busanjin-gu, Busan 614-714, Republic of Korea; b Department of Chemistry, Pukyong National University, 599-1 Daeyeon 3-dong, Nam-gu, Busan 608-737, Republic of Korea

**Keywords:** crystal structure, benzo­furan, 4-fluoro­phen­yl, π–π inter­actions, C—S⋯π and C—H⋯O inter­actions

## Abstract

In the title compound, C_17_H_14_ClFO_2_S, the dihedral angle between the mean planes of the benzo­furan ring system [maximum deviation = 0.037 (2) Å] and the 4-fluoro­benzene ring is 71.92 (5)°. An intra­molecular C—H⋯O hydrogen bond occurs. In the crystal, mol­ecules are linked by π–π stacking between the benzene rings of neighbouring mol­ecules [centroid–centroid distance = 3.7103 (10) Å]. These mol­ecules are further linked by C—S⋯π [S⋯centroid = 3.570 (1) Å] and C—H⋯O inter­actions, resulting in a three-dimensional supra­molecular network.

## Related literature   

For the pharmacological properties of benzo­furan compounds, see: Aslam *et al.* (2009 ▶); Galal *et al.* (2009 ▶); Howlett *et al.* (1999 ▶); Khan *et al.* (2005 ▶); Ono *et al.* (2002 ▶). For natural products with a benzo­furan ring, see: Akgul & Anil (2003 ▶); Soekamto *et al.* (2003 ▶). For the synthesis of the starting material 5-chloro-3-(4-fluoro­phenyl­sulfan­yl)-2,4,6-trimethyl-1-benzo­furan, see: Choi *et al.* (1999 ▶). For a related structure, see: Choi *et al.* (2012 ▶).
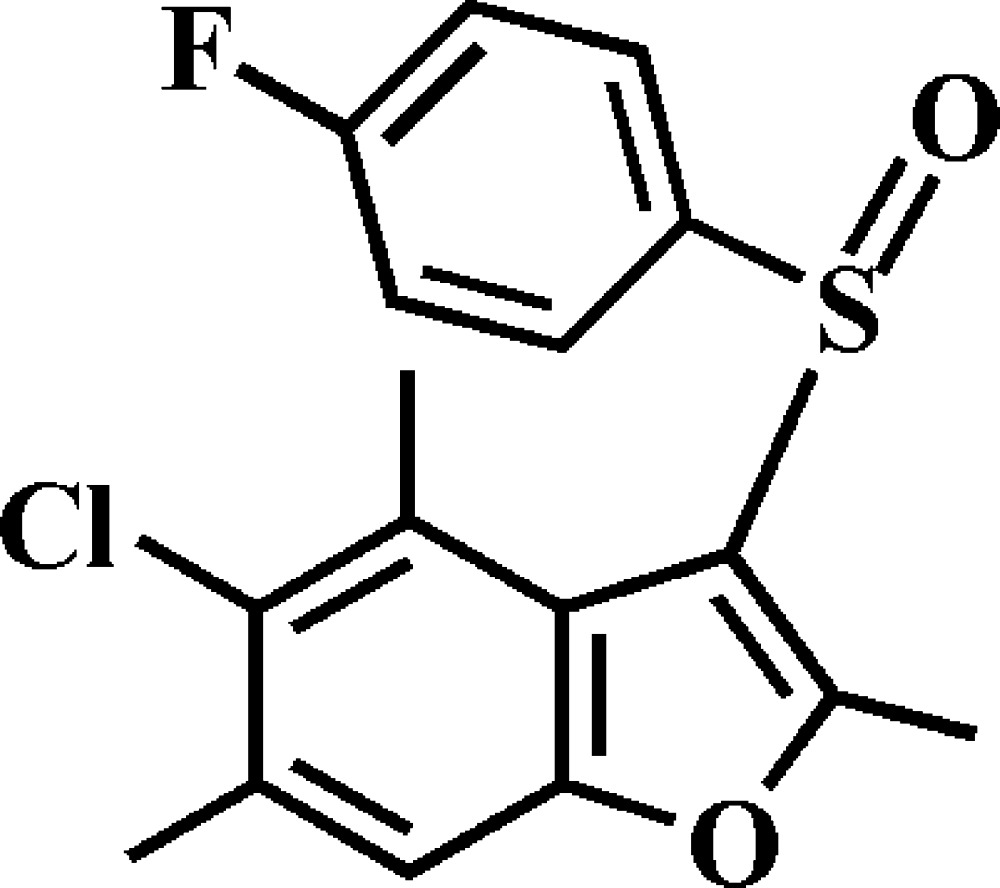


## Experimental   

### Crystal data  

C_17_H_14_ClFO_2_S
*M* *_r_* = 336.79
Monoclinic, 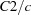

*a* = 21.7503 (3) Å
*b* = 10.6444 (2) Å
*c* = 16.4061 (3) Å
β = 126.622 (1)°
*V* = 3048.49 (9) Å^3^
*Z* = 8
Mo *K*α radiation
μ = 0.40 mm^−1^
*T* = 173 K
0.43 × 0.26 × 0.25 mm

### Data collection  

Bruker SMART APEXII CCD diffractometer
Absorption correction: multi-scan (*SADABS*; Bruker, 2009 ▶) *T*_min_ = 0.846, *T*_max_ = 0.908
14427 measured reflections
3777 independent reflections
3158 reflections with *I* > 2σ(*I*)
*R*_int_ = 0.030

### Refinement  

*R*[*F* ^2^ > 2σ(*F* ^2^)] = 0.037
*wR*(*F* ^2^) = 0.096
*S* = 1.06
3777 reflections
202 parameters
H-atom parameters constrained
Δρ_max_ = 0.27 e Å^−3^
Δρ_min_ = −0.38 e Å^−3^

### 

Data collection: *APEX2* (Bruker, 2009 ▶); cell refinement: *SAINT* (Bruker, 2009 ▶); data reduction: *SAINT*; program(s) used to solve structure: *SHELXS97* (Sheldrick, 2008 ▶); program(s) used to refine structure: *SHELXL97* (Sheldrick, 2008 ▶); molecular graphics: *ORTEP-3 for Windows* (Farrugia, 2012 ▶) and *DIAMOND* (Brandenburg, 1998 ▶); software used to prepare material for publication: *SHELXL97*.

## Supplementary Material

Crystal structure: contains datablock(s) global, I. DOI: 10.1107/S1600536814019229/xu5819sup1.cif

Structure factors: contains datablock(s) I. DOI: 10.1107/S1600536814019229/xu5819Isup2.hkl

Supporting information file. DOI: 10.1107/S1600536814019229/xu5819Isup3.cml

**Fig. 1.**
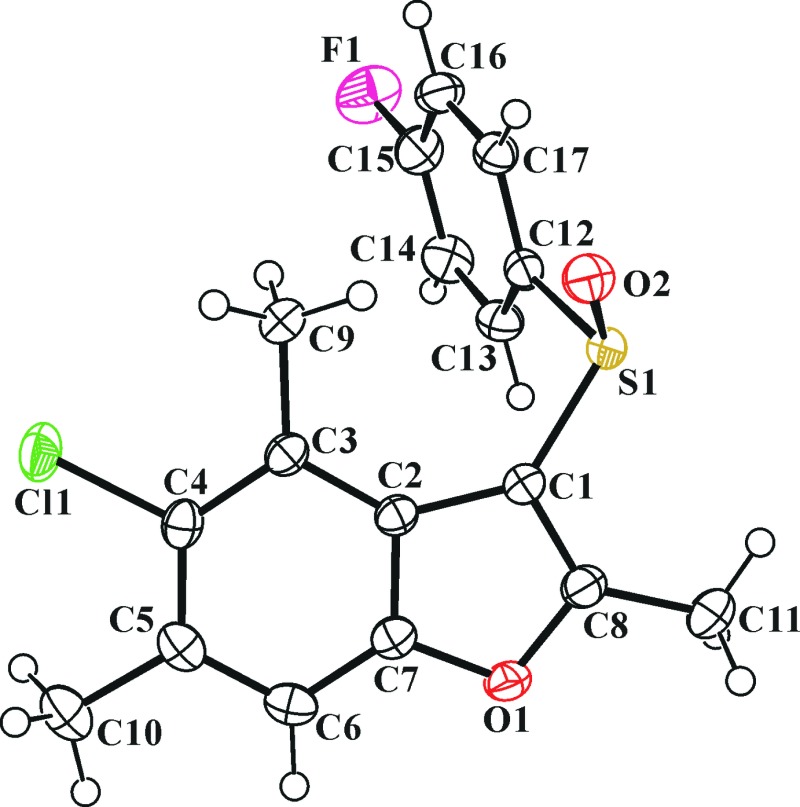
The molecular structure of the title compound with the atom numbering scheme. Displacement ellipsoids are drawn at the 50% probability level. H atoms are presented as small spheres of arbitrary radius.

. DOI: 10.1107/S1600536814019229/xu5819fig1.tif
The mol­ecular structure of the title compound with the atom numbering scheme. Displacement ellipsoids are drawn at the 50% probability level. H atoms are presented as small spheres of arbitrary radius.

**Fig. 2.**
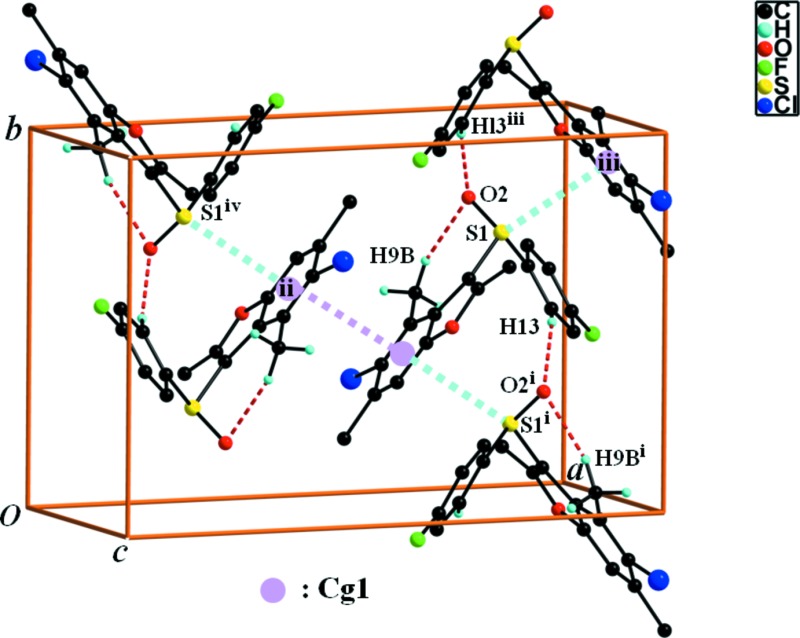
A view of the C—H···O, C—S···π and π···π interactions (dotted lines) in the crystal structure of the title compound. H atoms non-participating in hydrogen-bonding were omitted for clarity. [Symmetry codes: (i) - x + 3/2, y - 1/2, - z + 3/2; (ii) - x + 1, - y + 1 , - z + 1; (iii) 1.5- x + 3/2, y + 1/2, - y + 3/2.]

Click here for additional data file.x y x y x y y . DOI: 10.1107/S1600536814019229/xu5819fig2.tif
A view of the C—H⋯O, C—S⋯π and π⋯π inter­actions (dotted lines) in the crystal structure of the title compound. H atoms non-participating in hydrogen-bonding were omitted for clarity. [Symmetry codes: (i) − *x* + 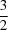, *y* − 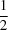, − z + 3/2; (ii) − *x* + 1, − *y* + 1, − z + 1; (iii) 1.5 − *x* + 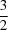, *y* + 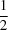, − *y* + 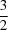.]

CCDC reference: 1021158

Additional supporting information:  crystallographic information; 3D view; checkCIF report

## Figures and Tables

**Table 1:** Hydrogen-bond geometry (Å, °)

*D*—H⋯*A*	*D*—H	H⋯*A*	*D*⋯*A*	*D*—H⋯*A*
C9—H9*B*⋯O2	0.98	2.45	3.3901 (19)	161
C13—H13⋯O2^i^	0.95	2.46	3.3191 (19)	150

Symmetry code: (i) 
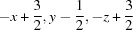
.

## supplementary crystallographic information

### S1. Comment

Benzofuran compounds show interesting biological activity such as antibacterial and antifungal, antitumor and antiviral, antimicrobial activities (Aslam *et al.* 2009, Galal *et al.*, 2009, Khan *et al.*, 2005), and potential inhibitor of β-amyloid aggregation (Howlett *et al.*, 1999, Ono *et al.*, 2002). These benzofuran compounds occur in a great number of natural products. (Akgul & Anil, 2003, Soekamto *et al.*, 2003). As a part of our ongoing study of 3-arylsulfinyl-5-chloro-2-methyl-1-benzofuran derivatives containing 4-bromophenylsulfinyl substituent in 3-position (Choi *et al.*, 2012), we report herein on the crystal structure of the title compound.

In the title molecule (Fig. 1), the benzofuran unit is essentially planar, with a mean deviation of 0.022 (1) Å from the least-squares plane defined by the nine constituent atoms. The 4-fluorophenyl ring is essentially planar, with a mean deviation of 0.007 (1) Å from the least-squares plane defined by the six constituent atoms. The dihedral angle formed by the benzofuran ring and the 4-fluorophenyl ring is 71.92 (5)°. In the crystal structure (Fig. 2), molecules are linked by π···π interactions between the benzene rings of neighbouring molecules, with a Cg1···Cg1^ii^ distance of 3.7103 (10) Å and an interplanar distance of 3.489 (1) Å resulting in a slippage of 1.261 (1) Å (Cg1 is the centroid of the C2–C7 benzene ring). These molecules are further linked by C—S···π interactions between the sulfur atom and the centroid of the benzene ring of an adjacent molecule with S1···Cg1^iii^ being 3.570 (1) Å, and by C—H···O hydrogen bonds (Table 1), resulting in a three-dimensional network.

### S2. Experimental

The starting material 5-chloro-3-(4-fluorophenylsulfanyl)-2,4,6-trimethyl-1-benzofuran was prepared by literature method (Choi *et al.* 1999). 3-Chloroperoxybenzoic acid (77%, 224 mg, 1.0 mmol) was added in small portions to a stirred solution of 5-chloro-3-(4-fluorophenylsulfanyl)-2,4,6-trimethyl-1-benzofuran (288 mg, 0.9 mmol) in dichloromethane (20 mL) at 273 K. After being stirred at room temperature for 10h, the mixture was washed with saturated sodium bicarbonate solution (2 X 10 mL) and the organic layer was separated, dried over magnesium sulfate, filtered and concentrated at reduced pressure. The residue was purified by column chromatography (hexane–ethyl acetate, 2:1 *v*/*v*) to afford the title compound as a colorless solid [yield 64% (215 mg); m.p. 432–433 K; *R*_f_ = 0.62 (hexane–ethyl acetate, 2:1 *v*/*v*)]. Single crystals suitable for X-ray diffraction were prepared by slow evaporation of a solution of the title compound (24 mg) in ethyl acetate (15 mL) at room temperature.

### S3. Refinement

All H atoms were positioned geometrically and refined using a riding model, with C—H = 0.95 Å for aryl and 0.98 Å for methyl H atoms, *U*_iso_ (H) = 1.2*U*_eq_(C) for aryl and 1.5*U*_eq_(C) for methyl H atoms. The positions of methyl hydrogens were optimized using the SHELXL-97 command AFIX 137 (Sheldrick, 2008).

### Crystal data

**Table:**

C_17_H_14_ClFO_2_S	*F*(000) = 1392
*M**_r_* = 336.79	*D*_x_ = 1.468 Mg m^−^^3^
Monoclinic, *C*2/*c*	Melting point = 433–432 K
Hall symbol: -C 2yc	Mo *K*α radiation, λ = 0.71073 Å
*a* = 21.7503 (3) Å	Cell parameters from 6565 reflections
*b* = 10.6444 (2) Å	θ = 2.6–28.3°
*c* = 16.4061 (3) Å	µ = 0.40 mm^−^^1^
β = 126.622 (1)°	*T* = 173 K
*V* = 3048.49 (9) Å^3^	Block, colourless
*Z* = 8	0.43 × 0.26 × 0.25 mm

### Data collection

**Table:**

Bruker SMART APEXII CCD diffractometer	3777 independent reflections
Radiation source: rotating anode	3158 reflections with *I* > 2σ(*I*)
Graphite multilayer monochromator	*R*_int_ = 0.030
Detector resolution: 10.0 pixels mm^-1^	θ_max_ = 28.3°, θ_min_ = 2.2°
φ and ω scans	*h* = −28→28
Absorption correction: multi-scan (*SADABS*; Bruker, 2009)	*k* = −14→13
*T*_min_ = 0.846, *T*_max_ = 0.908	*l* = −17→21
14427 measured reflections	

### Refinement

**Table:**

Refinement on *F*^2^	Primary atom site location: structure-invariant direct methods
Least-squares matrix: full	Secondary atom site location: difference Fourier map
*R*[*F*^2^ > 2σ(*F*^2^)] = 0.037	Hydrogen site location: difference Fourier map
*wR*(*F*^2^) = 0.096	H-atom parameters constrained
*S* = 1.06	*w* = 1/[σ^2^(*F*_o_^2^) + (0.0482*P*)^2^ + 1.6356*P*] where *P* = (*F*_o_^2^ + 2*F*_c_^2^)/3
3777 reflections	(Δ/σ)_max_ = 0.001
202 parameters	Δρ_max_ = 0.27 e Å^−^^3^
0 restraints	Δρ_min_ = −0.38 e Å^−^^3^

### Special details

**Table:**

Experimental. ^1^H NMR (δ p.p.m., CDCl_3_, 400 Hz): 7.42-7.49 (m, 2H), 7.21 (s, 1H), 7.11-7.17 (m, 2H), 2.72 (s, 3H), 2.44 (s, 3H), 2.31 (s, 3H).
Geometry. All esds (except the esd in the dihedral angle between two l.s. planes) are estimated using the full covariance matrix. The cell esds are taken into account individually in the estimation of esds in distances, angles and torsion angles; correlations between esds in cell parameters are only used when they are defined by crystal symmetry. An approximate (isotropic) treatment of cell esds is used for estimating esds involving l.s. planes.
Refinement. Refinement of F^2^ against ALL reflections. The weighted R-factor wR and goodness of fit S are based on F^2^, conventional R-factors R are based on F, with F set to zero for negative F^2^. The threshold expression of F^2^ > 2sigma(F^2^) is used only for calculating R-factors(gt) etc. and is not relevant to the choice of reflections for refinement. R-factors based on F^2^ are statistically about twice as large as those based on F, and R- factors based on ALL data will be even larger.

### Fractional atomic coordinates and isotropic or equivalent isotropic displacement parameters (Å^2^)

**Table:**

	*x*	*y*	*z*	*U*_iso_*/*U*_eq_	
Cl1	0.53070 (2)	0.33359 (4)	0.37985 (3)	0.03606 (13)	
S1	0.74572 (2)	0.73246 (4)	0.72173 (3)	0.02365 (11)	
F1	0.93275 (7)	0.44638 (11)	0.63815 (9)	0.0507 (3)	
O1	0.64319 (6)	0.49502 (10)	0.78458 (8)	0.0269 (2)	
O2	0.70024 (6)	0.82105 (10)	0.63429 (8)	0.0292 (3)	
C1	0.68686 (8)	0.61394 (14)	0.71580 (11)	0.0226 (3)	
C2	0.63706 (8)	0.51802 (14)	0.64153 (11)	0.0213 (3)	
C3	0.61055 (8)	0.48595 (14)	0.54264 (11)	0.0221 (3)	
C4	0.56460 (8)	0.37943 (14)	0.50245 (11)	0.0241 (3)	
C5	0.54266 (8)	0.30586 (15)	0.55238 (12)	0.0265 (3)	
C6	0.56660 (8)	0.34393 (15)	0.64791 (12)	0.0266 (3)	
H6	0.5515	0.2996	0.6836	0.032*	
C7	0.61300 (8)	0.44806 (14)	0.68942 (11)	0.0232 (3)	
C8	0.68791 (9)	0.59471 (15)	0.79844 (11)	0.0262 (3)	
C9	0.63008 (9)	0.56232 (16)	0.48421 (11)	0.0283 (3)	
H9A	0.6735	0.5237	0.4901	0.042*	
H9B	0.6438	0.6478	0.5118	0.042*	
H9C	0.5857	0.5654	0.4126	0.042*	
C10	0.49526 (10)	0.18871 (17)	0.50524 (15)	0.0374 (4)	
H10A	0.4903	0.1467	0.5542	0.056*	
H10B	0.5203	0.1320	0.4862	0.056*	
H10C	0.4443	0.2111	0.4445	0.056*	
C11	0.72805 (11)	0.65747 (18)	0.89850 (13)	0.0383 (4)	
H11A	0.6906	0.6839	0.9099	0.057*	
H11B	0.7556	0.7312	0.8998	0.057*	
H11C	0.7646	0.5988	0.9521	0.057*	
C12	0.80080 (8)	0.63903 (14)	0.69532 (11)	0.0225 (3)	
C13	0.83516 (9)	0.52796 (15)	0.74711 (12)	0.0284 (3)	
H13	0.8277	0.4970	0.7949	0.034*	
C14	0.88046 (10)	0.46287 (16)	0.72827 (13)	0.0331 (4)	
H14	0.9048	0.3866	0.7629	0.040*	
C15	0.88957 (10)	0.51084 (16)	0.65836 (13)	0.0327 (4)	
C16	0.85635 (9)	0.62098 (16)	0.60663 (12)	0.0299 (4)	
H16	0.8635	0.6511	0.5583	0.036*	
C17	0.81203 (9)	0.68691 (14)	0.62708 (11)	0.0255 (3)	
H17	0.7895	0.7648	0.5942	0.031*	

### Atomic displacement parameters (Å^2^)

**Table:**

	*U*^11^	*U*^22^	*U*^33^	*U*^12^	*U*^13^	*U*^23^
Cl1	0.0410 (2)	0.0357 (3)	0.0314 (2)	−0.00531 (17)	0.0216 (2)	−0.01340 (17)
S1	0.0279 (2)	0.0221 (2)	0.0255 (2)	−0.00352 (14)	0.01845 (17)	−0.00428 (14)
F1	0.0588 (7)	0.0477 (7)	0.0658 (8)	0.0197 (6)	0.0481 (7)	0.0053 (6)
O1	0.0290 (6)	0.0332 (6)	0.0228 (5)	−0.0028 (5)	0.0177 (5)	0.0011 (4)
O2	0.0361 (6)	0.0221 (6)	0.0356 (6)	0.0038 (5)	0.0247 (5)	0.0023 (5)
C1	0.0241 (7)	0.0236 (8)	0.0232 (7)	−0.0011 (6)	0.0158 (6)	−0.0016 (6)
C2	0.0220 (7)	0.0216 (7)	0.0220 (7)	0.0020 (6)	0.0140 (6)	0.0010 (6)
C3	0.0233 (7)	0.0228 (7)	0.0234 (7)	0.0034 (6)	0.0156 (6)	−0.0003 (6)
C4	0.0235 (7)	0.0235 (8)	0.0238 (7)	0.0035 (6)	0.0133 (6)	−0.0032 (6)
C5	0.0205 (7)	0.0224 (8)	0.0329 (8)	0.0016 (6)	0.0139 (7)	0.0004 (6)
C6	0.0229 (7)	0.0268 (8)	0.0309 (8)	0.0027 (6)	0.0165 (7)	0.0072 (6)
C7	0.0221 (7)	0.0253 (8)	0.0229 (7)	0.0030 (6)	0.0138 (6)	0.0019 (6)
C8	0.0268 (8)	0.0301 (8)	0.0242 (7)	−0.0009 (6)	0.0166 (7)	−0.0006 (6)
C9	0.0332 (8)	0.0314 (9)	0.0252 (8)	−0.0028 (7)	0.0202 (7)	−0.0030 (6)
C10	0.0341 (9)	0.0292 (9)	0.0456 (10)	−0.0074 (7)	0.0220 (8)	−0.0049 (8)
C11	0.0463 (10)	0.0478 (11)	0.0248 (8)	−0.0100 (8)	0.0234 (8)	−0.0070 (7)
C12	0.0221 (7)	0.0221 (7)	0.0233 (7)	−0.0024 (6)	0.0136 (6)	−0.0026 (6)
C13	0.0301 (8)	0.0269 (8)	0.0295 (8)	−0.0008 (6)	0.0185 (7)	0.0035 (6)
C14	0.0339 (9)	0.0256 (8)	0.0384 (9)	0.0065 (7)	0.0209 (8)	0.0071 (7)
C15	0.0331 (9)	0.0316 (9)	0.0404 (9)	0.0038 (7)	0.0256 (8)	−0.0032 (7)
C16	0.0355 (9)	0.0308 (9)	0.0324 (8)	−0.0001 (7)	0.0251 (8)	0.0014 (7)
C17	0.0294 (8)	0.0223 (8)	0.0270 (8)	0.0000 (6)	0.0181 (7)	0.0012 (6)

### Geometric parameters (Å, º)

**Table:**

Cl1—C4	1.7487 (15)	C9—H9A	0.9800
S1—O2	1.4943 (11)	C9—H9B	0.9800
S1—C1	1.7581 (16)	C9—H9C	0.9800
S1—C12	1.7977 (15)	C10—H10A	0.9800
F1—C15	1.3543 (19)	C10—H10B	0.9800
O1—C8	1.3637 (19)	C10—H10C	0.9800
O1—C7	1.3767 (18)	C11—H11A	0.9800
C1—C8	1.358 (2)	C11—H11B	0.9800
C1—C2	1.458 (2)	C11—H11C	0.9800
C2—C7	1.391 (2)	C12—C17	1.378 (2)
C2—C3	1.402 (2)	C12—C13	1.386 (2)
C3—C4	1.390 (2)	C13—C14	1.382 (2)
C3—C9	1.499 (2)	C13—H13	0.9500
C4—C5	1.406 (2)	C14—C15	1.374 (2)
C5—C6	1.385 (2)	C14—H14	0.9500
C5—C10	1.505 (2)	C15—C16	1.373 (2)
C6—C7	1.375 (2)	C16—C17	1.386 (2)
C6—H6	0.9500	C16—H16	0.9500
C8—C11	1.481 (2)	C17—H17	0.9500
			
O2—S1—C1	110.89 (7)	H9A—C9—H9C	109.5
O2—S1—C12	105.80 (7)	H9B—C9—H9C	109.5
C1—S1—C12	99.07 (7)	C5—C10—H10A	109.5
C8—O1—C7	106.37 (11)	C5—C10—H10B	109.5
C8—C1—C2	106.89 (13)	H10A—C10—H10B	109.5
C8—C1—S1	118.35 (12)	C5—C10—H10C	109.5
C2—C1—S1	134.60 (11)	H10A—C10—H10C	109.5
C7—C2—C3	119.32 (14)	H10B—C10—H10C	109.5
C7—C2—C1	104.18 (12)	C8—C11—H11A	109.5
C3—C2—C1	136.49 (14)	C8—C11—H11B	109.5
C4—C3—C2	115.50 (13)	H11A—C11—H11B	109.5
C4—C3—C9	122.47 (13)	C8—C11—H11C	109.5
C2—C3—C9	122.03 (13)	H11A—C11—H11C	109.5
C3—C4—C5	125.13 (14)	H11B—C11—H11C	109.5
C3—C4—Cl1	117.63 (12)	C17—C12—C13	121.44 (15)
C5—C4—Cl1	117.24 (12)	C17—C12—S1	117.25 (12)
C6—C5—C4	117.84 (14)	C13—C12—S1	121.15 (12)
C6—C5—C10	120.25 (15)	C14—C13—C12	119.09 (15)
C4—C5—C10	121.90 (15)	C14—C13—H13	120.5
C7—C6—C5	117.75 (14)	C12—C13—H13	120.5
C7—C6—H6	121.1	C15—C14—C13	118.54 (15)
C5—C6—H6	121.1	C15—C14—H14	120.7
C6—C7—O1	124.54 (14)	C13—C14—H14	120.7
C6—C7—C2	124.32 (14)	F1—C15—C16	117.87 (16)
O1—C7—C2	111.12 (13)	F1—C15—C14	118.86 (15)
C1—C8—O1	111.43 (13)	C16—C15—C14	123.27 (15)
C1—C8—C11	133.34 (15)	C15—C16—C17	117.94 (15)
O1—C8—C11	115.22 (13)	C15—C16—H16	121.0
C3—C9—H9A	109.5	C17—C16—H16	121.0
C3—C9—H9B	109.5	C12—C17—C16	119.69 (15)
H9A—C9—H9B	109.5	C12—C17—H17	120.2
C3—C9—H9C	109.5	C16—C17—H17	120.2
			
O2—S1—C1—C8	−126.73 (13)	C8—O1—C7—C2	−0.91 (16)
C12—S1—C1—C8	122.41 (13)	C3—C2—C7—C6	3.0 (2)
O2—S1—C1—C2	58.56 (17)	C1—C2—C7—C6	−177.73 (14)
C12—S1—C1—C2	−52.31 (16)	C3—C2—C7—O1	−178.47 (13)
C8—C1—C2—C7	−0.45 (17)	C1—C2—C7—O1	0.84 (16)
S1—C1—C2—C7	174.69 (13)	C2—C1—C8—O1	−0.09 (18)
C8—C1—C2—C3	178.67 (17)	S1—C1—C8—O1	−176.16 (10)
S1—C1—C2—C3	−6.2 (3)	C2—C1—C8—C11	178.60 (18)
C7—C2—C3—C4	−3.6 (2)	S1—C1—C8—C11	2.5 (3)
C1—C2—C3—C4	177.39 (16)	C7—O1—C8—C1	0.60 (17)
C7—C2—C3—C9	175.93 (14)	C7—O1—C8—C11	−178.34 (14)
C1—C2—C3—C9	−3.1 (3)	O2—S1—C12—C17	20.45 (14)
C2—C3—C4—C5	1.2 (2)	C1—S1—C12—C17	135.32 (13)
C9—C3—C4—C5	−178.27 (14)	O2—S1—C12—C13	−164.15 (12)
C2—C3—C4—Cl1	−179.26 (11)	C1—S1—C12—C13	−49.28 (14)
C9—C3—C4—Cl1	1.2 (2)	C17—C12—C13—C14	−1.2 (2)
C3—C4—C5—C6	2.0 (2)	S1—C12—C13—C14	−176.44 (12)
Cl1—C4—C5—C6	−177.49 (11)	C12—C13—C14—C15	−0.2 (2)
C3—C4—C5—C10	−177.52 (15)	C13—C14—C15—F1	−178.81 (16)
Cl1—C4—C5—C10	3.0 (2)	C13—C14—C15—C16	0.5 (3)
C4—C5—C6—C7	−2.8 (2)	F1—C15—C16—C17	179.87 (15)
C10—C5—C6—C7	176.77 (15)	C14—C15—C16—C17	0.5 (3)
C5—C6—C7—O1	−177.99 (14)	C13—C12—C17—C16	2.3 (2)
C5—C6—C7—C2	0.4 (2)	S1—C12—C17—C16	177.70 (12)
C8—O1—C7—C6	177.66 (14)	C15—C16—C17—C12	−1.9 (2)

### Hydrogen-bond geometry (Å, º)

**Table:**

*D*—H···*A*	*D*—H	H···*A*	*D*···*A*	*D*—H···*A*
C9—H9*B*···O2	0.98	2.45	3.3901 (19)	161
C13—H13···O2^i^	0.95	2.46	3.3191 (19)	150

Symmetry code: (i) −*x*+3/2, *y*−1/2, −*z*+3/2.
