# Supplementary figures and images for: Crystal structure of 5-chloro-3-(4-fluoro­phenyl­sulfin­yl)-2,4,6-trimethyl-1-benzo­furan
Source: Acta Crystallogr Sect E Struct Rep Online. 2014 Sep 3;70(Pt 10):o1078–9. doi: 10.1107/S1600536814019229 (PMC4257165; doi:10.1107/S1600536814019229)

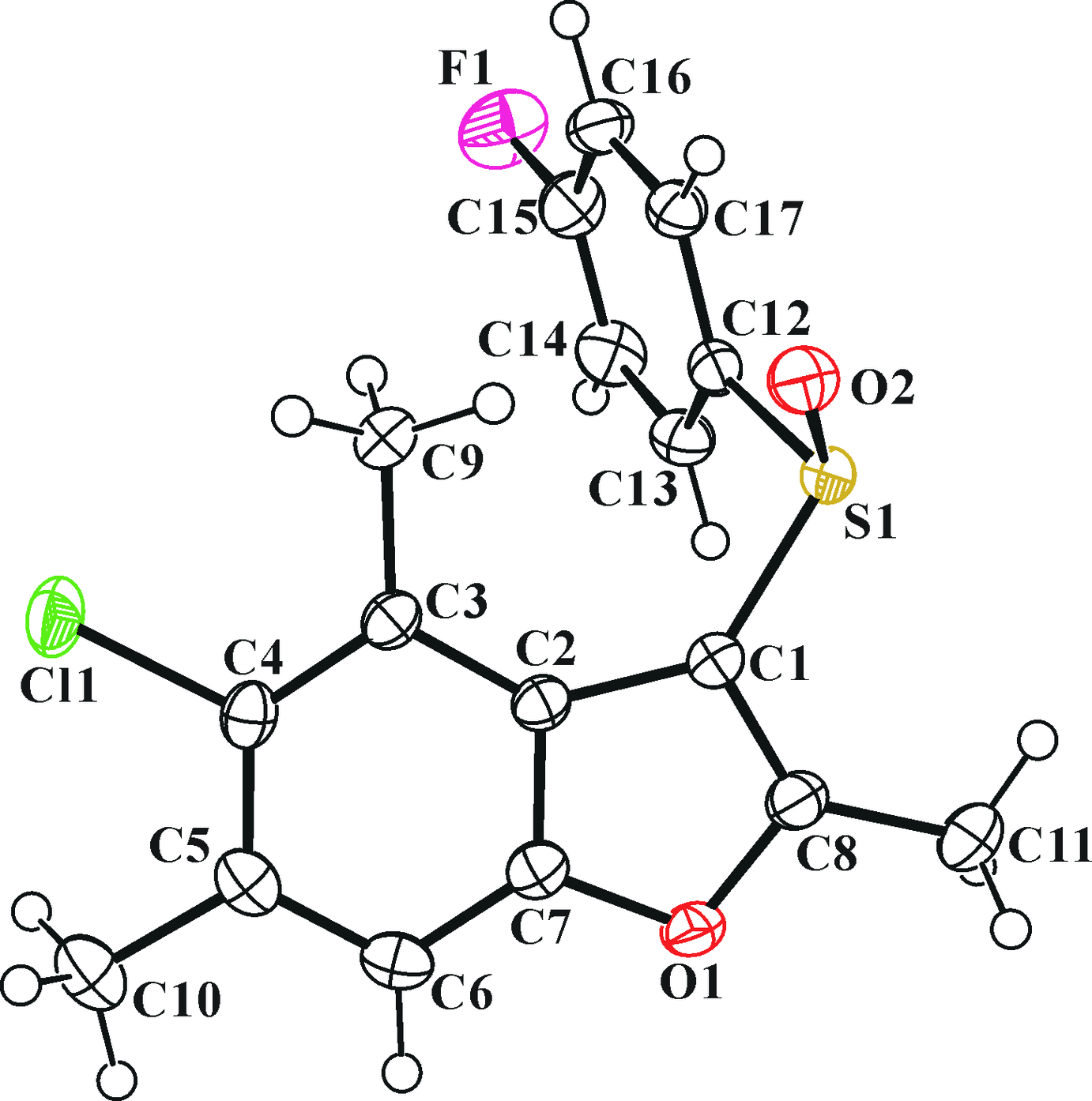

Supplement: Supplementary file 4 [file e-70-o1078-fig1.tif]

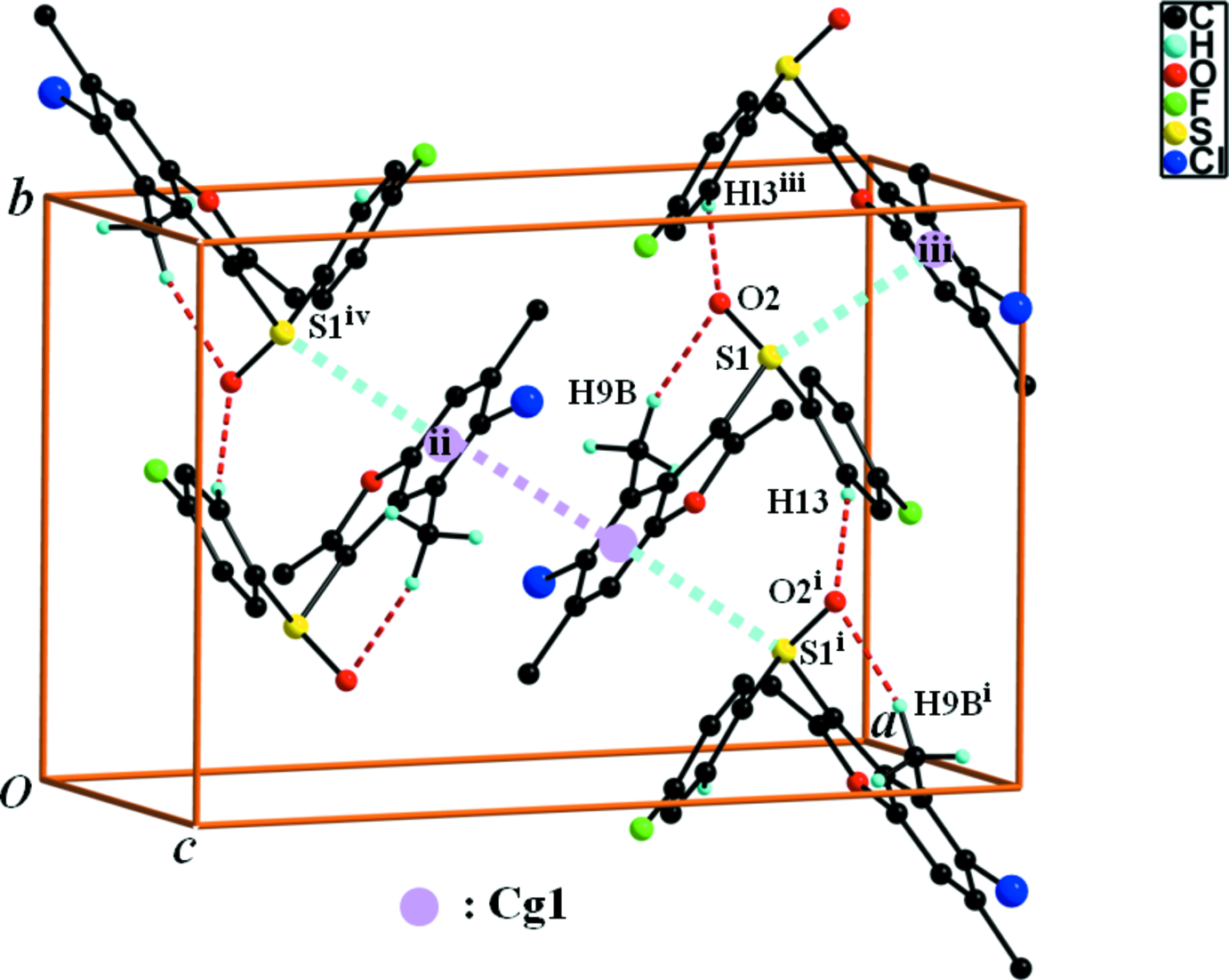

Supplement: Supplementary file 5 [file e-70-o1078-fig2.tif]
